# Ethnic disparities in the epidemiology, treatment, and outcome of patients with hepatocellular carcinoma in the United States

**DOI:** 10.20517/2394-5079.2023.10

**Published:** 2023-05-18

**Authors:** Mohammad Saeid Rezaee-Zavareh, Jeff Liang, Ju Dong Yang

**Affiliations:** 1 Middle East Liver Diseases Center, Tehran 1417935840, Iran.; 2 Department of Internal Medicine, Cedars-Sinai Medical Center, Los Angeles 90048, CA, USA.; 3 Comprehensive Transplant Center, Cedars-Sinai Medical Center, Los Angeles 90048, CA, USA.; 4 Karsh Division of Gastroenterology and Hepatology, Cedars-Sinai Medical Center, Los Angeles 90048, CA, USA.; 5 Samuel Oschin Comprehensive Cancer Institute, Cedars-Sinai Medical Center, Los Angeles 90048, CA, USA.

**Keywords:** Hepatocellular Carcinoma, ethnic, disparity

## Abstract

There are significant ethnic disparities in incidence, tumor stage, curative therapy receipt, and survival among patients with hepatocellular carcinoma (HCC) in the US. While previous models had predicted an increasing trend in the incidence rate of HCC until 2030 in the US, recent studies have shown that HCC incidence plateaued in 2013 and then started to decline in 2015. The decreasing trend has been observed in all ethnicities except for American Indians/Alaska Natives, whose incidence rates of HCC continue to rise. Current evidence shows that African-Americans and Hispanics are two groups that are more likely to be diagnosed with late-stage HCC, and this finding has been consistent in different socioeconomic statuses of the patients. These two ethnic minority groups are also among those who are less likely to have curative therapy for early-stage HCC. Finally, advances in early diagnosis and treatment approaches have led to an improvement in HCC survival for all ethnicities; however, African-Americans continue to have the worst survival. More studies to find the causes of these disparities and interventions to eliminate them are urgently needed.

## HEPATOCELLULAR CARCINOMA BURDEN AND RISK FACTORS

Hepatocellular carcinoma (HCC), which accounts for 75% to 85% of primary liver cancers, is one of the leading causes of cancer incidence and mortality in the world and the US^[1,2]^. The incidence and mortality of liver cancer are estimated to increase by 55% and 56.4%, respectively, from 2020 to 2040^[3]^. Several factors contribute to the high mortality rate of HCC. First, patients with early-stage HCC are usually asymptomatic; thus, the majority of patients are diagnosed in the late stage with poor clinical outcomes^[4]^. Second, HCC has a high rate of recurrence even after curative treatment for early-stage diseases^[5]^, which leads to a high incidence-to-mortality ratio.

HCC risk factors include heavy consumption of alcohol, chronic hepatitis B (HBV) and hepatitis C (HCV) virus infection, cigarette smoking, type 2 diabetes, obesity, non-alcoholic fatty liver disease (NAFLD), exposure to environmental toxins such as foods containing aflatoxin, and genetics^[3,6,7]^. Currently, alcohol is considered the leading cause of cirrhosis worldwide and the incidence of alcohol-associated hepatitis has increased in recent years^[8]^. There has been a shift in the etiology of HCC over the past decades^[9]^. HCC burden from viral etiologies has been reduced by highly effective interventions such as the HBV vaccine and antiviral treatment, including direct-acting antivirals (DAAs). Conversely, the rising rates of obesity and diabetes have increased the incidence of NASH cirrhosis as a risk factor for HCC^[10–12]^.

The etiology, incidence rate, and mortality of HCC vary by country and ethnicity^[1,6,13]^. Differences in access to routine healthcare, surveillance, diagnostic testing, and treatment likely contribute to these disparities^[14]^. In this commentary, we aim to summarize the ethnic disparities in HCC incidence, treatment, and survival in the US.

## DISCRETE PATTERN IN THE HCC INCIDENCE TREND

Over the past few decades, there has been a concerning trend in the incidence of hepatocellular carcinoma (HCC) in the United States. Initially, from 2000 to 2007, there was a considerable increase with an annual percentage change (APC) of 5.64% (*P* < 0.001), followed by a significant continued increase from 2007 to 2013 with an APC of 2.68% (*P* < 0.001). However, the APC plateaued around 2013 with a value of −1.44% (*P* = 0.12)^[15]^, and then there has been a significant decline in HCC incidence from 2015 to 2017 (APC: −3.5, *P*: 0.02)^[16]^. These incidence trends differ by ethnicity. Studies using the Surveillance, Epidemiology, and End Results Program (SEER) and National Program of Cancer Registries (NPCR) databases show that from 2000–2015, HCC had the highest incidence among Asians/Pacific Islanders (APIs), American Indians/Alaska Natives (AI/ANs) and Hispanics, and lower incidence in African-Americans and Whites^[16,17]^. In 2018, the age-standardized incidence rate for HCC was highest among American Indian/Alaska Natives (AI/ANs) at 9.27 per 100,000 person-years. The corresponding rates for Hispanics, African-Americans, APIs, and Whites were 8.88, 8.21, 8.08, and 4.29, respectively^[18]^.

In 2015–2018, the overall incidence of HCC decreased, and rates among APIs and Whites followed a similar trend^[17]^. APIs had the highest incidence of HCC in the US previously, a finding that has been attributed to the high prevalence of HBV infection among immigrants from endemic regions^[19,20]^. Due to HBV vaccine programs and effective antiviral therapies, the HCC incidence rate among API stabilized, and then began decreasing significantly in 2007 (APC: −2.72%, *P* < 0.001), and is predicted to continue declining until 2030 with an estimated APC of −1.59% −2.20% in men and women, respectively^[15,20–22]^. The incidence rates among Hispanics and African-Americans have remained stagnant^[15]^. In contrast, the incidence of HCC in AI/ANs significantly increased from 2015 to 2018 (APC: 4.3%)^[18]^. This may be related to the high disease burden of HCV and diabetes/metabolic syndrome in AI/ANs. Type 2 diabetes has the highest prevalence (> 15%) among AI/ANs compared to other ethnic groups in the US^[23]^. In 2013, the mortality rate of HCV among AI/ANs was reported to be more than twice the national average, with 12.2 deaths per 100,000 people compared to 5.0 per 100,000, respectively^[24]^. Particularly, it has shown that among AI/ANs with chronic HCV infection, those infected with genotype-3 HCV have a greater than threefold increased risk of developing HCC compared to patients infected with genotype-1 HCV (hazard ratio [HR]: 3.06, 95% confidence interval [CI]: 1.43–6.55)^[25]^. Screening and access to HCV treatment and hepatology care are challenging in this population due to socioeconomic/cultural barriers, which may contribute to the steady increase in HCC incidence^[24,26]^.

Country of origin likely contributes to intra-ethnic variations in HCC incidence. Among Hispanic men, the age-adjusted incidence rate of HCC was almost twice as high in US-born compared to foreign-born individuals, although a similar pattern was not observed in Hispanic women (44.7 *vs.* 23.1 and 14.5 *vs.* 13.4 per 100,000 people for men and women, respectively)^[27]^. These findings support the presence of disparate exposures to HCC risk factors among Hispanic men born in the US. While the prevalence of metabolic syndrome and alcohol use has been found to be higher in Hispanic patients, it is unclear whether these risk factors are more prevalent among US-born Hispanics compared to foreign-born ones, and additional studies are needed to elucidate these exposures^[27]^.

## TUMOR STAGE BY DIFFERENT ETHNICITY

Multiple studies from the US found that African-American patients were more likely to be diagnosed with late-stage disease with larger tumor size, independent of socioeconomic status [Table 1].

From 2008–2017, African-Americans were 26% more likely to be diagnosed with late-stage HCC compared to Whites^[33]^. A recent meta-analysis of 20 articles with 209,622 HCC patients found that while there were no disparities in APIs or Hispanics compared to Whites (odds ratio[OR]: 1.01, 95%CI: 0.97–1.05), African-Americans have a 34% (OR: 0.66, 95%CI: 0.54–0.78) lower chance of early tumor detection than Whites^[13]^. Lower frequency of HCC surveillance among African-Americans^[36,37]^, community economic disadvantage^[35]^, and differences in etiology of liver disease and detection of cirrhosis^[33]^ likely contribute to these findings. Intra-ethnical differences in HCC staging have also been found-among APIs, Filipinos were more likely than Chinese to be diagnosed with late-stage HCC^[32]^.

## CURATIVE TREATMENT RECEIPT BY DIFFERENT ETHNICITY

African-Americans and Hispanics are less likely than other ethnicities to undergo curative treatment for HCC [Table 2].

A retrospective study using SEER, evaluating more than 60,000 HCC patients during 1998–2010, showed that compared with White HCC patients, African-Americans, APIs, and Hispanics have 52%, 35%, and 24% lower odds of undergoing liver transplantation^[39]^. Another study with the SEER database showed that APIs had the greatest odds of receiving any form of treatment, in particular surgery^[40]^. Ha *et al*. showed that among patients with HCC within Milan criteria, APIs were 20% more likely to receive curative treatment than Whites, although APIs were less likely to undergo liver transplantation. The lower likelihood of liver transplant surgery in APIs is likely attributed to a higher proportion of patients having non-cirrhotic liver disease or well-compensated cirrhosis from HBV etiology, making them an ideal candidate for surgical resection compared to liver transplant. The authors also showed that Hispanics and African-Americans were less likely to receive curative therapy compared to Whites^[29]^.

Investigation of databases other than SEER, including Indiana University Academic Medical Center (2000–2014)^[31]^, Parkland Memorial Health and Hospital System, and UT Southwestern Medical Center (2008–2017)^[33]^, have reported the same disparity in treatment for Hispanics and African-Americans. Additionally, five other databases and registries showed that between 2000–2014, Hispanics were significantly less likely to undergo resection or liver transplantation compared to Whites^[34]^. Interestingly, disparities between Whites and African-Americans in receiving curative therapy have been reported even among insured people^[42]^. A study of 3990 HCC patients using the US National Cancer Database showed that Hispanics and African-Americans were significantly less likely to receive immunotherapy compared with White patients for advanced-stage HCC, likely due to differential access to clinical trials and experimental therapies. The results suggest that ethnic disparity is not limited to curative treatment, but remains pervasive in the entire spectrum of HCC treatment^[43]^.

The reasons for this disparity are multifaceted and may involve various interacting factors. The growth pattern of tumors in HCC can be influenced by the underlying etiology causing the HCC. For instance, a retrospective study conducted from 2008 to 2017 on cirrhotic patients with HCC found that the indolent tumor growth type was more common in nonviral cirrhosis (50.9%) than viral cirrhosis (32.1%)^[44]^. Additionally, the distribution of HCC underlying disorders has been reported to vary among different ethnic groups. A study among HCC cases in the US showed that between 2000 and 2011, the population attributable fractions of metabolic disorders were highest among Hispanics (39.3%) and Whites (34.8%), while the largest population attributable fractions for HCV were reported for African-Americans (36.1%) and Asians (29.7%)^[45]^. Therefore, different ethnic groups may also exhibit distinct tumor growth patterns.

Groups with different tumor growth patterns and underlying etiologies may also have different socioeconomic statuses^[42]^, access to healthcare facilities, and surveillance rates^[37,46]^. A retrospective study aimed to characterize HCC surveillance among 904 cirrhotic patients between 2008 and 2011^[46]^. The study found that 67% of patients had inconsistent surveillance, defined as having at least one screening ultrasound in a 3-year period. The study demonstrated that African American patients had a 39% lower chance of having inconsistent surveillance (OR: 0.61, 95%CI: 0.42–0.99). The study also found that insurance status and having multiple primary care and hepatology visits per year significantly increased the chance of inconsistent surveillance. Additionally, low socioeconomic status has been found to be associated with a lower rate of curative treatment receipt for HCC^[36]^, with the effect being more pronounced among African-Americans. A retrospective study that examined nearly 14,000 HCC patients aged 65 years or older between 2000–2015 found that African-Americans living in high-poverty neighborhoods were 36% less likely to receive curative treatment compared to Whites with similar socioeconomic status (OR: 0.64, 95%CI: 0.49–0.84)^[42]^. However, socioeconomic status did not have a significant effect on curative treatment receipt among Asians and Hispanics in this study. Additionally, implicit or explicit biases can also play a role in health disparities by affecting access to healthcare and the quality of care received^[47]^. All these factors are believed to contribute to the ethnic disparities in the receipt of curative treatments.

## OVERALL SURVIVAL

Multiple studies evaluated ethnic disparities in the survival of HCC patients, most of which found a significantly shorter survival time among African-Americans. For instance, one study of more than 6,000 liver transplant recipients for HCC during 2002–2013 reported African-American ethnicity as an independent risk factor for shorter 5-year overall survival^[48]^. A recent meta-analysis found that African-Americans with HCC had a 1.08-fold higher mortality rate (HR: 1.08, 95%CI: 1.05–1.12) compared to Whites. In contrast, Hispanics and Asians had an 8% (HR: 0.92, 95%CI: 0.87–0.97) and 19% (HR: 0.81, 95%CI: 0.73–0.88) better survival rate compared to Whites, respectively^[13]^.

A SEER-based study examining newly diagnosed HCC from 1994 to 2011 found that APIs have more favorable outcomes than Whites. In this study, treatment characteristics appeared to have a more important role than tumor and patient characteristics^[49]^. Among Asians, the highest overall five-year survival has been reported for Chinese [33.1 %, 95%CI: (30.3–45.8)] and the lowest for Japanese [22.0 %, 95%CI: (18.2–26.2)] and Filipinos [19.9 %, 95%CI: (16.8–23.1)]. In other words, compared to Chinese, Japanese and Filipinos have had a 1.48 (*P* < 0.001) and 1.57 (*P* < 0.001) significantly higher risk of mortality, respectively ^[32]^.

The “Hispanic paradox,” which describes how Hispanic patients have comparable^[34]^ or better^[50]^ survival rates than Whites and African-Americans in spite of living in areas with low socioeconomic status, has been observed among HCC cases. This phenomenon may be attributed to geography, as Hispanic patients are more likely to reside in close proximity to academic centers, which gives them greater access to multidisciplinary care^[50]^. The survival advantage observed in Hispanic patients with HCC may also be partly attributed to differences in demographics and the etiology of underlying liver disease. For instance, a higher proportion of Hispanic HCC patients are women, and they are more likely to have NAFLD^[13,51]^.

The overall survival of HCC patients has significantly improved due to advances in early detection of HCC cases and treatment modalities^[52]^. While ethnical disparities still exist, these advances have helped narrow some of the differences. In a cohort of more than 20,000 HCC patients between 2004 and 2015, African-American ethnicity has been identified as a significant risk factor contributing to a worse prognosis. However, this study also showed a considerable improvement in the five-year overall survival of HCC patients from 21.99% to 29.85% for the years 2004 and 2012, respectively^[53]^. A different study examining the HCC survival outcomes from 2002–2017 found that African-Americans had the greatest increase in survival rates during this time period, although their overall survival remained lower than other ethnicities^[54]^. The availability of DAA for HCV treatment is considered one of the major factors contributing to improved HCC survival in African-American patients^[54,55]^.

## CONCLUSION

Striking ethnic disparities exist in epidemiology, management, and outcome of HCC patients in the US. In the HCC care continuum, low implementation of HCC screening rates with different levels of access to healthcare facilities and subspecialties could contribute to ethnic disparity in early detection of HCC. Various HCC etiology, tumor biology, host genetic and socioeconomic factors among different ethnicity likely contribute to the variation in curative treatment receipt and outcome. Furthermore, our understanding of the interaction between HCC etiology and host genetics is still incomplete. It is essential to further investigate these interactions to better understand the mechanisms underlying the ethnic disparities in HCC outcomes. Finally, comprehensive interventions are needed on different levels in the cascade of care to decrease these ethnic disparities and achieve health equity in HCC patients.

## Figures and Tables

**Table 1.** Hepatocellular stage at presentation by different ethnicity

First Author	Evaluated Database and Registry	Study Period	Comparator	Main related Results

Sloane^[28]^	(SEER)-11, Alaska database	1999–2001	Whites	More regional and distant metastasis at diagnosis for African-Americans
Ha^[29]^	SEER, United Network of Organ Sharing	2003–2011	Whites	More advanced HCC at diagnosis for African-Americans and less advanced disease for Asians
Franco^[30]^	SEER	2000–2012	Whites	More metastasis at diagnosis for African-Americans
Dakhoul^[31]^	Indiana University Academic Medical Center	2000–2014	Whites	Similar results for African-Americans regarding Barcelona Clinic Liver Cancer (BCLC) staging and Milan criteria A trend for having larger tumor size (*P* = 0.05)
_Yu_^[32]^	SEER	2004–2012	Chinese Asians	More advanced disease for Filipinos
_Rich_^[33]^	Parkland Memorial Health and Hospital System, UT Southwestern Medical Center	2008–2017	Whites	More diagnoses at a late stage for African-Americans and Hispanics
Pomenti^[34]^	Five United States academic medical centers (Atrium Health, Columbia University Irving Medical Center, Indiana University Health Hospital, MD Anderson Cancer Center, and Vanderbilt University Medical Center)	2000–2014	Whites	Larger tumor size, more advanced stage, high rate of microvascular invasion for Hispanics at presentation
Flores^[17]^	SEER-18	2000–2015	AI/ANs, APIs, Hispanics, Whites	More proportion with late-stage HCC for African-Americans in both low and high socioeconomic status
Oluyomi^[35]^	Texas Cancer Registry	2007–2015	Whites	More distant stage for Non-Hispanic African-Americans and more local stage for Hispanics

SEER: Surveillance, Epidemiology, and End Results; AI/ANs: Americans Indians/Alaska/Natives; APIs: Asians/Pacific Islanders

**Table 2.** Curative Treatment Receipt by Different Ethnicity

First Author	Evaluated Database and Registry	Study Period	Comparator	Main related Results

_Zak_^[38]^	California Cancer Registry	1996–2006	Whites	Less chance of undergoing liver transplantation, ablation, or hepatectomy for African-Americans and Hispanics. The ethnicity effect has been decreased by considering the status of insurance and socioeconomic of patients
_Wong_^[39]^	SEER	1998–2010	Whites	Less chance of undergoing liver transplantation for African-Americans, Asians, and Hispanics
_Xu_^[40]^	SEER	1998–2012	Whites, AfricanAmericans, Native Americans	Greatest chance of receiving any form of curative treatment, in particular, surgery for Asians
_Ha_^[29]^	SEER, United Network of Organ Sharing	2003–2011	NHWs	More chance of receiving curative treatment for Asians and less chance for African-Americans and Hispanics. Less chance of undergoing liver transplantation for Asians and Hispanics.
Dakhoul^[31]^	Indiana University Academic Medical Center	2000–2014	Whites	Less chance of undergoing liver transplantation for African-Americans
_Rich_^[33]^	Parkland Memorial Health and Hospital System and UT Southwestern Medical Center	2008–2017	Whites	Less chance of receiving curative treatment for Hispanics
Scaglione^[41]^	Four health systems: 2 tertiary care centers (University of Michigan and Loyola University) and 2 safety-net health systems (Parkland Health and Hospital System and Ben Taub Hospital-Harris Health System)	2012–2013	-	Ethnicity has no effect on the curative treatment receipt after considering the health system and insurance
Pomenti^[34]^	Five United States academic medical centers (Atrium Health, Columbia University Irving Medical Center, Indiana University Health Hospital, MD Anderson Cancer Center, and Vanderbilt University Medical Center)	2000–2014	NHWs	Less chance of undergoing resection or liver transplantation
Wagle^[42]^	SEER	2001–2015	Whites	Similar chance of receiving curative treatment for Hispanics and Asians in all socioeconomic levels and less chance for African-Americans living in high-poverty neighborhoods

SEER: Surveillance, Epidemiology, and End Results; NHW: non-Hispanic Whites; AI/ANs: Americans Indians/Alaska/Natives; APIs: Asians/Pacific Islanders

## Data Availability

Not applicable.
